# Immune Microenvironment Signatures as Biomarkers to Predict Early Recurrence of Stage Ia-b Lung Cancer

**DOI:** 10.3389/fonc.2021.680287

**Published:** 2021-07-28

**Authors:** Qiang Wang, Danting Zhou, Fang Wu, Qingchun Liang, Qiongzhi He, Muyun Peng, Tianyu Yao, Yan Hu, Banglun Qian, Jingqun Tang, Xiang Wang, Wenliang Liu, Fenglei Yu, Chen Chen

**Affiliations:** ^1^Department of Thoracic Surgery, The Second Xiangya Hospital of Central South University, Changsha, China; ^2^Hunan Key Laboratory of Early Diagnosis and Precise Treatment of Lung Cancer, The Second Xiangya Hospital of Central South University, Changsha, China; ^3^Department of Oncology, The Second Xiangya Hospital of Central South University, Changsha, China; ^4^Department of Pathology, The Second Xiangya Hospital of Central South University, Changsha, China; ^5^Geneplus-Beijing Institute, Beijing, China

**Keywords:** lung cancer, recurrence, TILs (tumor-infiltrating lymphocytes), gene expression, biomarker

## Abstract

**Introduction:**

Approximately 30% of patients diagnosed with stage Ia-b NSCLC die of recurrent disease after surgery. This study aimed to identify immune-related biomarkers that might predict tumor recurrence in stage Ia-b NSCLC within 40 months after curative resection.

**Methods:**

Gene expression data of stage Ia-b NSCLC samples was retrieved from the TCGA database, the GEO databases, and the Second Xiangya hospital (XXEYY) database. 22 types of tumors infiltrating immune cells and the expression of immune-associated genes were investigated using CIBERSORT, immunohistochemical staining, and GSEA analyses in a total of 450 patients (80 in the training cohort and 370 in the validation cohorts). Recurrence-related immune features were selected based on the LASSO Cox regression model.

**Results:**

High density of Tregs, Macrophages M0 and M1 cell could be observed in recurrence group while the memory B cell was more frequently enriched in controls, yet Tregs alone was significantly associated with tumor early recurrence in TCGA cohort, XYEYY cohort and GSE37745 dataset. A handful of immune-related genes were identified in the recurrence group. Based on Lasso regression analysis, the expressions of five immune-related genes, RLTPR, SLFN13, MIR4500HG, HYDIN and TPRG1 were closely correlated with tumor early recurrence. In the training cohort (TCGA), the combination of these five genes has sensitivity and specificity of 85% and 85%, with AUC of 0.91 (95% CI 0.84-0.98) for lung cancer early recurrence prediction, whereas in validation cohorts, the sensitivity and specificity using this panel was 61-89% and 54-82%, with AUC of 0.62-0.84.

**Conclusion:**

Our study demonstrated that the immune microenvironment signatures were closely related to tumor early recurrence. Compared to tumor-infiltrating lymphocytes, the expression of five immune-related genes could be robust biomarkers to predict early recurrence of stage Ia-b NSCLC after curative resection.

## Introduction

Lung cancer remains the leading global cause of cancer-related mortality (1). It is responsible for more than 1.6 million deaths every year and accounts for nearly one-third of all cancer deaths worldwide (2). Non-small cell lung cancer (NSCLC), which mainly includes adenocarcinoma, squamous cell carcinoma, and large cell carcinoma, constitutes the vast majority of lung cancer cases (more than 80%) (3). Curative surgical resection remains the most commonly employed treatment for patients with early-stage NSCLC. However, approximately 30% of patients diagnosed with stage Ia-b NSCLC die of recurrent disease after curative resection (4, 5). Most of these recurrences are systemic, such as lung, brain, liver, and bone cancers, indicating the existence of occult cancer cells far beyond the margins of surgical resection at the time of surgery (6, 7).

In the recent guidelines for lung cancer screening issued by National Comprehensive Cancer Network, low-dose computed tomography (CT) screening was recommended for high-risk lung cancer patients (8, 9). It is anticipated that increasing numbers of patients will be diagnosed with early-stage lung cancer, and nearly 30% of these patients will experience disease recurrence within five years after curative surgery (10, 11). Thus, there is a greater emphasis on identifying novel biomarkers that have high predicting value on the recurrence of early-stage NSCLC.

The prognostic utilities of molecular biomarkers, such as gene mutation, DNA methylation, and immune signatures, have been investigated for NSCLC (12, 13). The most frequently described genetic mutations within NSCLC involve mutations of KRAS(8-24%), EGFR(10-15%), BRAF(2-10%), ERBB2(1-2%), and PIK3CA(1-7%) (13–15). Despite the association between gene mutation and patient survival described in previous studies, as well as observations that many patients with certain gene mutational status have benefited from personalized target therapy (16), the correlation between gene mutation and disease recurrence remains unclear.

Previous studies have described the immune signatures were closely related to clinical outcomes in patients with malignant tumors (17, 18). Tumor-infiltrating lymphocytes (TILs) form an ecosystem in the tumor microenvironment to regulate cancer development and progression and have shown potential prognostic values (19). A high level of activated CD8^+^ T cells is associated with a better prognosis in many types of cancers, including NSCLC (20). Cytotoxic CD8^+^ T cells and CD4^+^ helper T cells could target antigenic tumor cells to suppress tumor progression. On the other hand, some tumor-infiltrating lymphocytes, such as regulatory T cells (Tregs), macrophages, and myeloid-derived suppressor cells (MDSCs), could inhibit T cell responses by secreting immunosuppressive cytokines or directly suppress T cell anti-tumor immunity, leading to immune escape (21). MDSCs were found to be critical factors in the formation of the premetastatic microenvironment after curative resection of primary NSCLC. A decreased accumulation of MDSCs in the premetastatic lung produces longer periods of disease-free survival and increased overall survival (22). Moreover, a particular group of immune-related genes has been identified as promising biomarkers for estimating overall survival in non-squamous NSCLC (23, 24). However, most of these studies observed various stages and used survival as the endpoint, diminishing their applicability in predicting the recurrence of early-stage NSCLC. In the present study, we investigated gene expression data of 450 stage Ia-b NSCLC cases from six independent cohorts to establish and validate a novel prognostic signature for NSCLC based on immune profiles. TILs and immune-related genes which were closely correlated with the early recurrence of stage Ia-b NSCLC were identified. The establishment of immune gene-based early recurrence biomarkers for patients with stage Ia-b NSCLC will help clarify the complex underlying mechanisms between immune responses and the recurrence of early-stage NSCLC and help optimize immunotherapies for patients with early-stage disease.

## Patients and Methods

### Publicly RNA Expression Datasets

TCGA RNA-seq data of samples from patients with Stage Ia-b NSCLC were acquired from the NCI’s Genomic Data Commons (GDC) (https://portal.gdc.cancer.gov/). All the patients were staged according to the 8th TNM guidelines classification criteria (25). The early recurrence group was defined as the patients with Stage Ia-b NSCLC in whom the tumor recurred within 40 months after curative surgery. While the controls were defined as the patients with Stage Ia-b NSCLC in whom there was no recurrence during the 40 months follow-up period (26). All of the patients had no macroscopically or microscopically positive surgical margins, and none of them received neoadjuvant chemotherapy or radiotherapy before surgery. Based on this including criteria, 34 patients from TCGA data were included in the recurrence group, and 46 patients were assigned to control group. The clinical data and recurrence-free survival (RFS) data were also collected (**Table 1**).

**Table 1:** Clinical characteristics of patients from TCGA cohort.

Patient Characteristics	Recurrence group (n = 34)	Control group (n = 46)	p-value
Age (year, IQR)	68 (48-88)	69 (56-83)	0.08
Gender (%)			
Male	16 (47.1%)	26 (56.5%)	0.40
Female	18 (52.9%)	20 (43.5%)	
Stage-no.(%)			
Ia	20 (58.8%)	21 (34.8%)	0.24
Ib	14 (41.2%)	25 (65.2%)	
Recurrence event type			N/A
Locoregional	14 (41.2%)	N/A	
Distant Metastasis	20 (58.8%)	N/A	
Gene mutation events-no*.			
EGFR	1 (9.0%)	2 (18.2%)	0.53
BRAF	3 (27.3%)	3 (27.3%)	1.00
ALK	0 (0.0%)	1 (9.0%)	0.31
AKT1	0 (0.0%)	0 (0.0%)	1.00
KRAS	3 (27.3%)	3 (27.3%)	1.00
HRAS	0 (0.0%)	0 (0.0%)	1.00
NRAS	0 (0.0%)	0 (0.0%)	1.00
MET	1(9.0%)	0 (0.0%)	0.31
ERBB2	1 (9.0%)	0 (0.0%)	0.31
ERBB4	1 (9.0%)	1 (9.0%)	1.00
MAP2K1	1 (9.0%)	1 (9.0%)	1.00
PIK3CA	1( 9.0%)	1 (9.0%)	1.00
STK11	1 (9.0%)	1 (9.0%)	1.00
Pack-Year (IQR)	47 (20-200)	40 (6-120)	0.50
FEV1/FVC (%, IQR)	72 (47-156)	69 (51-115)	0.11

*the gene mutation information could be found in 22 patients in the TCGA cohort, 11 in the recurrence group and 11 in the control group.

N/A, not available.

Based on the same including criteria, microarray data of patients with Stage Ia-b NSCLC from GSE31210, GSE116959, GSE32863, and GSE37745 were downloaded with complete clinical data using Gene Expression Omnibus (GEO) (http://www.ncbi.nlm.nih.gov/geo) to serve as validation datasets. GEO mRNA expression data was first log2 transformed and quantile normalized, then genes detected with more than one probe were calculated by mean expression. The clinical characteristics of the patients included in our study were shown in **Supplemental Table S1**.

### Patients and Fresh Frozen Tissue Samples From XYEYY Cohort

From October 2011 to June 2016, a total of 128 patients with NSCLC who underwent surgical resection at the Department of Thoracic Surgery, the Second Xiangya Hospital of Central South University were enrolled in this study (XYEYY cohort), including 42 patients in recurrence group and 86 patients in control group. All patients were staged to Stage Ia-b according to the 8th TNM guidelines classification criteria (25), including the histologic status of regional lymph nodes that were resected en bloc with the tumor and the mediastinal lymph nodes sampled from levels II, IV, VII, VIII, and IX on the right side and levels IV, V, VI, VII, VIII, and IX on the left side. All of the patients had no macroscopically or microscopically positive surgical margins. The clinic characteristics of the patients were collected from clinical notes, including demographics, smoking history, pulmonary function tests, and co-morbidities. Former smokers were defined as those individuals who had quit smoking within 15 years of the time of the study. Pack-years of cigarette smoking were defined as the average number of packs smoked per day multiplied by the number of years of smoking. Nodule size was obtained from the pathological report. None of the patients received neoadjuvant chemotherapy or radiotherapy before surgery. A summary of clinical and pathological characteristics of patients included in this study is presented in **Supplemental Table S1**.

This study was approved by the Institutional Review Board of the Second Xiangya Hospital Central South University and the requirement for informed consent was waived due to the study’s retrospective nature.

### RNA Extraction

All fresh tissue samples were washed with distilled deionized water and preserved using liquid nitrogen for quick freezing. The RNA extraction of tumor specimens was performed using the RNeasy Mini Kit (Qiagen), according to the manufacturer’s recommendations. RNA quantity and purity were measured with the NanoDrop ND-1000 spectrophotometer (Thermo Scientific). RNA integrity, determined by the RNA integrity number (RIN), was determined with the 2100 Bioanalyzer (Agilent).

### RNA-Seq Analysis

The detection and analysis of RNA-Seq were performed as described previously (27). Pathway enrichment analysis was based on Gene Set Enrichment Analysis (GSEA) (http://www.gsea-msigdb.org/gsea/index.jsp). Pathways with p-value<5% and q-value<25% are considered significantly enriched in differentially expressed or modified genes.

### Data Analysis

The TCGA dataset was adopted as the training set, which the GEO datasets and XYEYY dataset were conducted as validation sets. All the FPKM (Fragments Per Kilobase Million) information was normalized by the GAPDH expression of corresponding samples before lasso logistic analysis.

### LASSO Logistic Analysis

A Least Absolute Shrinkage and Selection Operator (LASSO) analysis were carried out to identify recurrence predictors. The recurrence signatures were further built based on the coefficients of LASSO logistic analysis. The sensitivity and specificity of the original model were obtained by the ROC curve. The performance of the model was internally validated and estimated by using the enhanced bootstrap method (n=100). The risk score for each patient was calculated using the linear combination of each expression of the gene multiplied by the LASSO coefficient (28).

### CIBERSORT Analysis

We used CIBERSORT (http://cibersort.stanford.edu/) to determine the relative fractions of 22 infiltrating immune cell types in each tumor tissue, with the algorithm run using the LM22 signature matrix at 1000 permutations. These Tumor-infiltrating immune cells included naïve B cells, memory B cells, plasma cells, naïve CD4^+^T cells, resting memory CD4^+^T cells, activated memory CD4^+^T cells, γδT cells, CD8^+^T cells, T follicular helper cells (Tfh), Tregs, macrophages (M1 macrophages, M2 macrophages, M0 macrophages), resting natural killer (NK) cells, activated NK cells, resting mast cells, activated mast cells, resting dendritic cells (DC), activated DC, monocytes, neutrophils and eosinophils. For each tumor sample, the sum of all evaluated immune cell type fractions equaled 1 (29–31).

### Quantitative Real-Time RT-PCR Analysis

Quantitative real-time RT-PCR was performed using a Prism 7500 Sequence Detection System (ABI, USA) and mRNA levels were quantified using the SYBR^®^Premix Ex Taq™(Takara Bio Inc., Dalian, China). All reactions were performed using at least two independent runs in triplicate each time. Then a dilution series of sample RNA was included to generate a standard curve used to calculate the relative concentrations of transcript present in each sample. Negative controls (in which water was substituted for RNA) were run for each sample. β-actin was also amplified and used as a loading control. As described previously, the 2^−ΔCt^ was calculated for each expression detection replicate comparing with the mean Ct for ACTB. For replicates that were not detected (ND), a Ct value of 100 was used, creating a near-zero value for 2^−ΔCt^. The mean 2^−ΔCt^ value and the cutoff were calculated with the formula previously (32, 33). Specific primers used for amplification were shown in **Supplemental Table S2**.

### IHC Analysis

IHC for CD4, CD8, and Foxp3 expression was performed as described in previous studies (34). Briefly, PEFF specimen blocks were cut into 4-μm-thick slices and mounted onto microscope slides. Rehydrated tissue sections were boiled in EDTA buffer (pH 8) with a microwave at 92°C for 30 minutes. After cooling down at room temperature (RT), tissue sections were successively incubated with a peroxidase block and a protein block for 10 minutes each. Sections were next incubated for 1h at RT with a mouse monoclonal anti-CD8/CD4/Foxp3 antibody (Abcam, Cambridge, UK). Sections were then incubated with EnVision anti-mouse HRP-conjugated antibody for 30 minutes at RT and then treated with the substrate diaminobenzidine tetrahydrochloride to allow visualization of the antigen-antibody complex. Lastly, the sections were lightly counterstained with hematoxylin. The percentages of positive lymphocytes as compared with the total amount of nucleated cells in the stromal compartments were assessed as previously described (35).

### Statistical Analyses

Demographic, gene expression and genotype variables were summarized by case-control status with percentages for categorical variables and means and standard deviations for continuous variables. Student’s t-tests or two-sided Wilcoxon rank-sum test was used to assessing p values between continuous variables, while Chi-squared tests or Fisher’s exact tests were used between two categorical variables. The association between the gene expression and case-control status were expressed as hazard ratios and their corresponding 95% confidence intervals obtained from logistic regression models with adjustment for the design variables (age and gender) and other important covariates, including smoking status, pack-years of smoking, and pulmonary function test results. Receiver operating curve (ROC) analysis was performed to investigate the performance of each individual gene in predicting cancer recurrence. The area under the curve (AUC) was reported with 95% confidence intervals (32, 33). All statistical analyses were performed by using SPSS 22.0 for Windows (SPSS, Chicago, IL, USA) and R 3.5.0 (http://www.r-project.org/). For all statistical analyses, a p-value< 0.05 was considered significant.

## Results

### Clinical Characteristics of the Patients

A total of 450 patients met inclusion criteria, with 151 recurrent cases and 299 controls in all datasets. According to the 8th edition of National Comprehensive Cancer Network Guidelines for the TNM classification for lung cancer (25), all of the subjects included in this study were patients with stage Ia-b NSCLC. The most frequent sites of recurrence were the ipsilateral lung, followed by metastasis to the brain, bone, mediastinum, and chest wall. Clinical and pathologic characteristics of patients in each dataset are shown in **Table 1** and **Supplemental Table S1**.

### Tumor-Infiltrating Lymphocytes Between Recurrence Group and Controls

**Figure 1:**
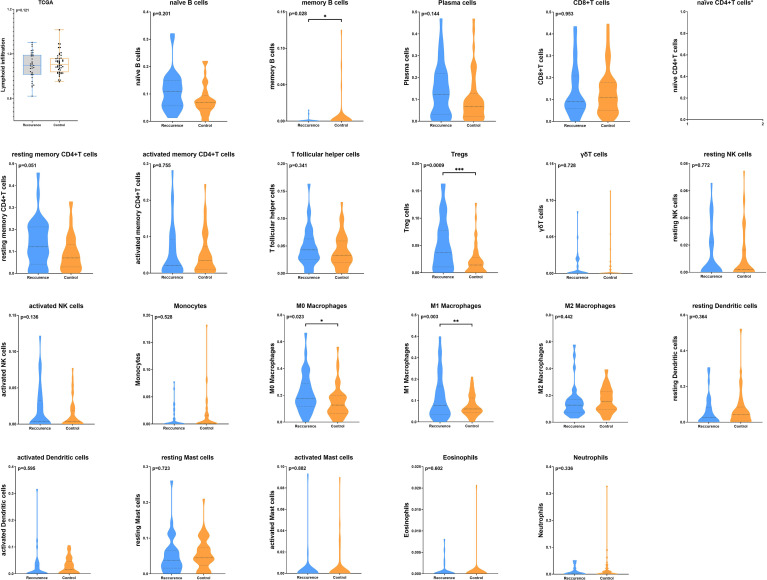
The distribution of infiltrating immune cells in the tumors from TCGA training cohort. CIBERSORT analysis demonstrated that the total number of infiltrated lymphocytes was similar between the two groups (p = 0.12). Among 22 tumor infiltrating lymphocyte types, Tregs, M0 and M1 macrophages significantly enriched in tumor tissues in the recurrence group, while memory B cells were more frequently detected in controls (*p < 0.05; **p < 0.01; ***p < 0.001). CIBERSORT analysis failed to generate the infiltration status of naïve CD4^+^T cells.

**Figure 2:**
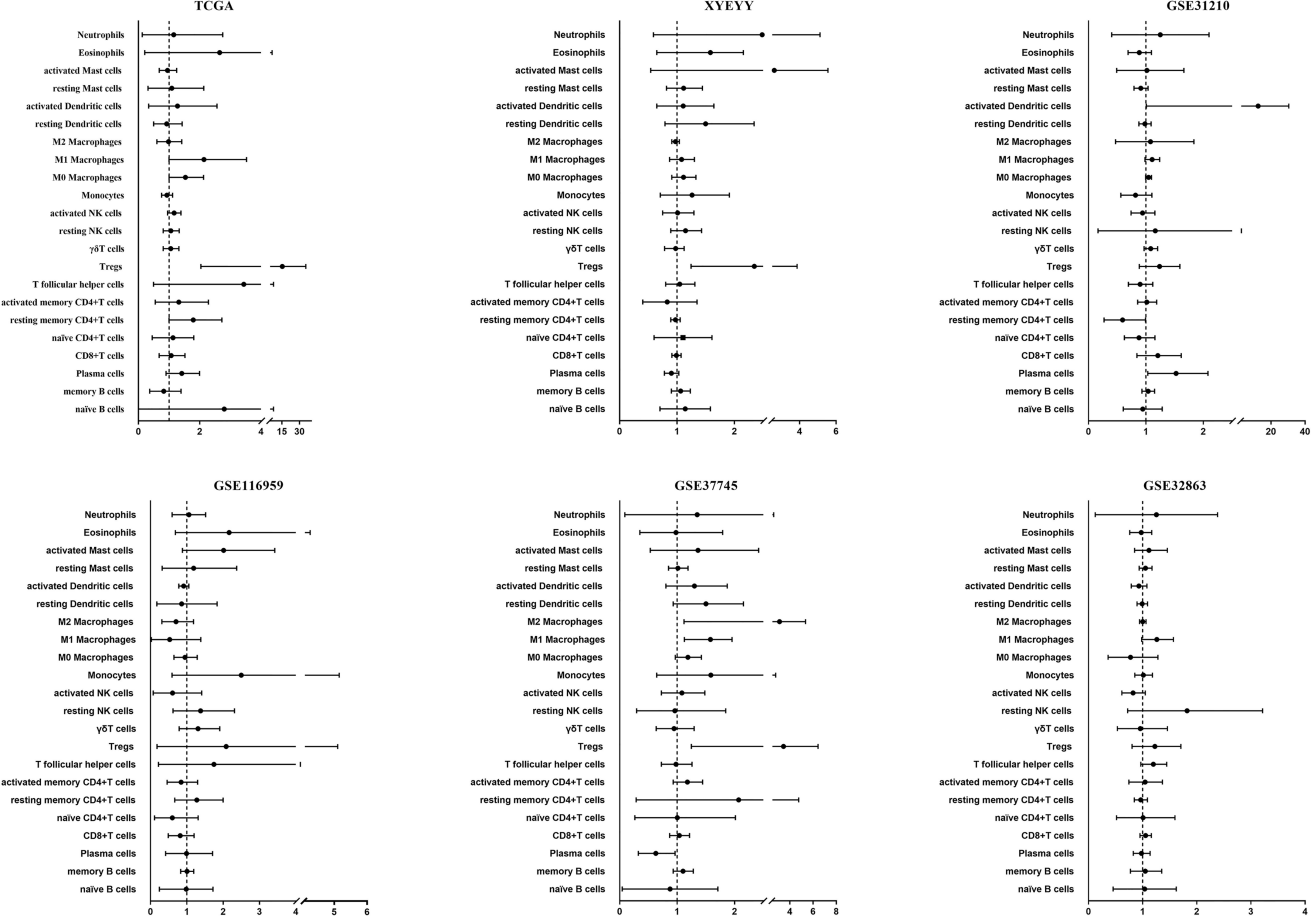
Performance of TILs as a predictor for lung cancer early recurrence. In the TCGA cohort, multivariate analysis revealed that the infiltration of Tregs was significantly related to tumor early recurrence (HR: 8.49, 95%CI: 2.04-35.44, p = 0.003). A strong correlation between Tregs infiltration and tumor early recurrence was also found in the XYEYY cohort (HR: 1.93, 95%CI: 1.25-3.86, p < 0.01) and GSE37745 dataset (HR: 2.68, 95%CI: 1.25-6.44, p < 0.01). The infiltration of Tregs lost its significance in GSE31210, GSE32863, and GSE116959 datasets.

We first investigated the distribution of infiltrating immune cells in the tumors from the TCGA training cohort. As shown in **Figure 1**, among 22 tumor-infiltrating lymphocyte types, Tregs, M0 and M1 macrophages significantly enriched in tumor tissues in the recurrence group, while memory B cells were more frequently detected in controls. Multivariate analysis revealed that the infiltration of Tregs was significantly related to tumor early recurrence (HR: 8.49, 95%CI: 2.04-35.44, p=0.003; **Figure 2**), which was consistent with Rooney and colleagues’ study (36). Next, we validate this result in XYEYY and GEO datasets. In the XYEYY cohort, CIBERSORT analysis demonstrated significant enrichment of Tregs in recurrence group, which were confirmed by IHC analysis (**Supplemental Figure S1**). A strong correlation between Tregs infiltration and tumor early recurrence was also found (HR: 1.93, 95%CI: 1.25-3.86, p<0.01; **Figure 2**). However, this correlation was only found in the GSE37745 dataset (HR: 2.68, 95%CI: 1.25-6.44, p<0.01; **Figure 2**), the infiltration of Tregs lost its significance in GSE31210, GSE32863, and GSE116959 datasets (**Figure 2**), indicating potential discordance when applying this biomarker in clinic practice.

### Association of Immune-Related Genes Expression and Tumor Early Recurrence

**Figure 3:**
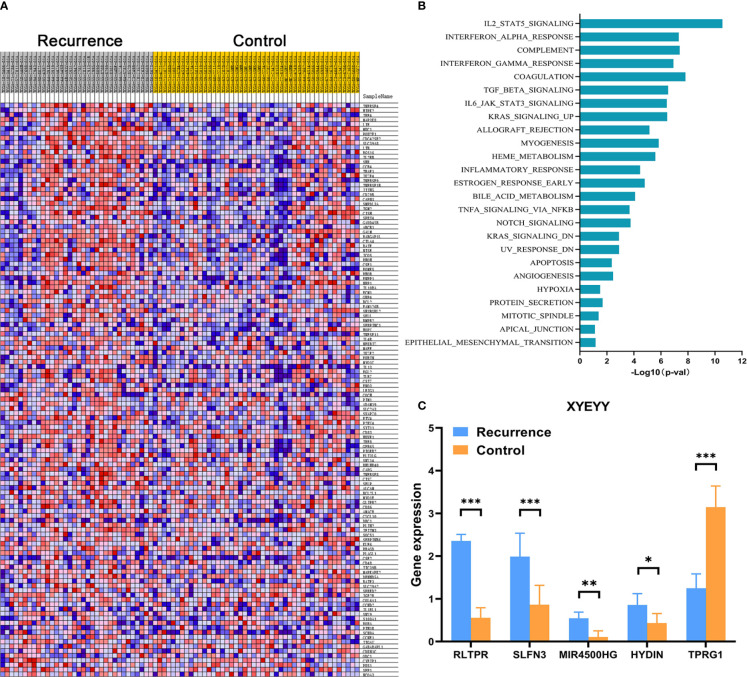
**(A)** Heatmap showed that, in the TCGA cohort, a certain number of immune-related genes were found to be overexpressed or down-regulated in the recurrence group compared to controls. **(B)** Several biological processes and pathways were identified to be related to these significant genes. **(C)** Quantitative real-time RT-PCR revealed the abnormal expression of RLTPR, SLFN13, HYDIN, MIR4500HG, and TPRG1 in the XYEYY cohort. *p < 0.05; **p < 0.01; ***p < 0.001.

In order to comprehensively assess the relationship between the expression of immune-related genes and tumor early recurrence, GSEA analyses were performed to identify differentially expressed genes between two groups. In the TCGA cohort, a certain number of immune-related genes were found to be overexpressed or down-regulated in recurrence group compared to controls (**Figure 3A**). Several biological processes and pathways were identified to be related to these significant genes (**Figure 3B**), which revealed that these genes were primarily involved in inflammatory pathways and innate immune responses. Further, a LASSO Cox regression model was used to calculate the most valuable prognostic genes, resulting in a model with five immune-related genes: RLTPR, SLFN13, HYDIN, MIR4500HG, and TPRG1.

**Figure 4:**
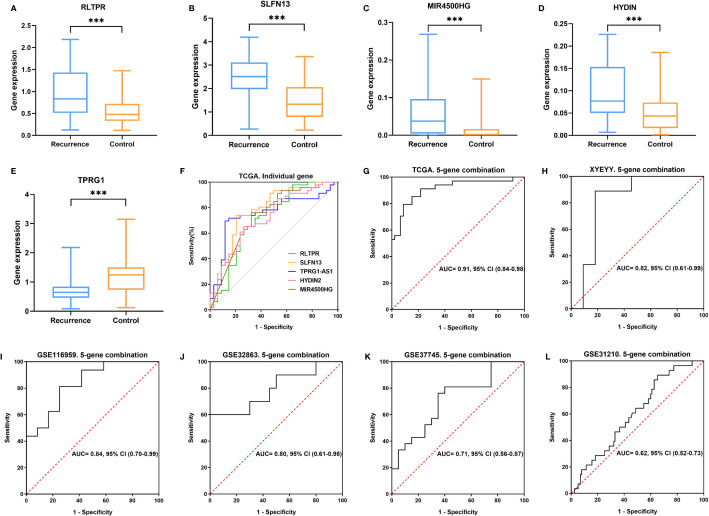
**(A**–**E)** In the TCGA cohort, the expression of RLTPR, SLFN13, HYDIN and MIR4500HG was higher in the recurrence group than in controls TPRG1 were significantly down-regulated in the recurrence group (***p < 0.001). **(F**, **G)** ROC curves for lung cancer early recurrence prediction using single gene and five-gene combination in TCGA cohort. **(H**–**L)** ROC curves for lung cancer early recurrence prediction using five-gene combination in XYEYY cohort and GEO datasets.

**Figure 5:**
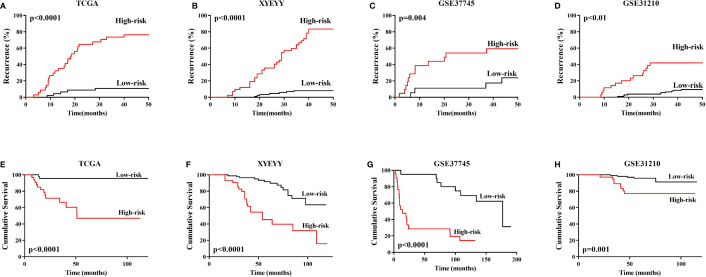
**(A–D)** In TCGA training cohort, XYEYY cohort and GEO datasets, Kaplan-Meier survival analyses showed that the rate of recurrence in the high-risk group were significantly higher than that in the low-risk group. **(E–H)** Consistently, the mortality rate in the high-risk group were significantly higher than that in low-risk group. Patients enrolled in GSE116959 dataset was conditioned by stringent criteria such as availability of resected surgical specimens, good quality RNA and time of follow-up for surviving patients (min 40 months for surviving patients). The recurrence and vital status were recorded, unfortunately, the time from surgery to recurrence or death were not available. Similarly, the recurrence status was recorded in GSE32863, but no time details. Thus Kaplan-Meier analyses cannot be performed in these two datasets.

As shown in **Figure 4**, in the TCGA cohort, the expression of RLTPR, SLFN13, HYDIN and MIR4500HG were higher in the recurrence group than that in the controls, while TPRG1 were significantly down-regulated in recurrence group (p<0.001). Based on the gene expression status, all the patients were assigned to the high-risk group and low-risk group. Kaplan-Meier survival analyses showed that the rate of recurrence in the high-risk group was significantly higher than that in the low-risk group. Consistently, the mortality rate in the high-risk group was significantly higher than that in the low-risk group (**Figures 5A, E**), indicating a strong correlation between gene expression and tumor recurrence-free survival.

To evaluate predictive performance of the five immune-related genes, ROC curves were obtained for each single gene using the normalized expression ΔCt values calculated as described in methods (32). There was high concordance between RLTPR (AUC: 0.71, 95%CI: 0.59-0.83), SLFN13(AUC: 0.78, 95%CI: 0.67-0.89), MIR4500HG (AUC: 0.74, 95%CI: 0.62-0.85), HYDIN (AUC: 0.72, 95%CI: 0.61-0.83) and TPRG1 (AUC: 0.75, 95%CI: 0.64-0.86) abnormal expressions and tumor early recurrence (**Figure 4F**). Multivariate analyses indicated that the expression of these five genes was closely related to increasing early recurrence risk (**Table 2**). We further evaluated the combination of the five genes. With sensitivity and specificity of 85% and 85%, the combination of these five genes have the largest AUC of 0.91 (95% CI 0.84-0.98) for lung cancer early recurrence prediction (**Figure 4G** and **Table 2**).

**Table 2:** Recurrence prediction using gene expression in TCGA training cohort.

Each gene in TCGA	Sensitivity	Specificity	AUC (95% CI)	HR	HR 95%CI	p-value
RLTPR	72%	65%	0.71 (0.59-0.83)	8.66	2.51-29.87	0.001
SLFN13	74%	79%	0.78 (0.67-0.89)	3.33	1.83-6.05	<0.001
MIR4500HG	76%	68%	0.74 (0.62-0.85)	3.68	1.59-8.51	0.002
HYDIN	65%	74%	0.72 (0.61-0.83)	2.16	1.37-3.42	0.001
TPRG1	74%	76%	0.75 (0.64-0.86)	0.16	0.06-0.47	0.001
**Five-gene combine**	85%	85%	0.91 (0.84-0.98)	19.18	6.36-57.85	<0.001

### Validation of the Five Gene Signature in the GEO Datasets

We validate the five-gene signature in four GEO datasets, GSE37745, GSE31210, GSE32863 and GSE116959. With similar gene expression status, patients were divided into high-risk group and low-risk group. Kaplan-Meier survival analyses revealed that patients in the low-risk group had significantly lower tumor early recurrence rates and better RFS than that in the high-risk group (**Figures 5C, D, G, H**), which were similar to the result from the TCGA cohort. We next validated the recurrence predictive performance of the combined five genes. At the best quantitative cutoff, the sensitivity and specificity of each GEO dataset ranged from 61% to 81% and 54% to 75%, respectively. The AUC of GSE116959, GSE37745, GSE31210 and GSE32863 were 0.84 (95% CI 0.70-0.99), 0.71 (95% CI 0.56-0.87), 0.62 (95% CI 0.52-0.73), 0.80 (95% CI 0.61-0.98), respectively (**Figures 4I–L**). With an elevated Hazard ratio from 1.88 to 8.23, the combination of five genes showed a strong association with tumor early recurrence (**Table 3**).

**Table 3:** The combination of five genes showed a strong association with tumor early recurrence in validation cohorts.

Cohorts	Sensitivity	Specificity	AUC (95% CI)	HR	HR 95%CI	p-value
XYEYY	89%	82%	0.82 (0.61-0.99)	7.91	3.34-35.9	<0.001
GSE116959	81%	75%	0.84 (0.70-0.99)	8.23	2.74-38.49	0.008
GSE37745	76%	65%	0.71 (0.56-0.87)	5.75	2.3-25.58	0.002
GSE32863	70%	70%	0.80 (0.61-0.98)	7.86	1.66-37.32	0.009
GSE31210	61%	54%	0.62 (0.52-0.73)	1.88	1.21-3.765	0.03

### Validation of the Five-Gene Signature in XYEYY Cohort From Frozen Tissue Samples

To evaluate the performance of the five-gene signature in predicting tumor early recurrence for patients with stage Ia-b NSCLC in clinic practice, we validated it in an independent cohort consisting of frozen tissue samples (**Supplemental Table S1**). Consistent with the TCGA cohort, abnormal expression of the five genes were also observed (**Figure 3C**). Based on the gene expression status, patients were also divided into high-risk group and low-risk group. High-risk patients had a significantly higher rate of early recurrence and shorter survival than the low-risk group (**Figures 5B, F**, p<0.0001). The AUC of the combined five genes was 0.82 (95% CI 0.61-0.99), with sensitivity and specificity of 89% and 82%, respectively (**Figure 4H**). Further regression analysis revealed that the five-gene signature was a significant risk factor for early tumor recurrence for patients with stage Ia-b NSCLC after curative surgical resection (HR: 7.91, 95% CI 3.34-35.9, p<0.001, **Table 3**).

## Discussion

Patients with early-stage NSCLC are at substantial risk for tumor recurrence and death, even after curative surgical resection. Due to a lack of consistent survival benefits, the use of adjuvant therapy in stage I NSCLC patients after surgery remains controversial. Identifying reliable prognostic biomarkers is critically needed to find out patients who are at high risk for early recurrence and who might benefit from additional systemic therapy. Recent studies revealed that prognostic biomarkers related to the tumor immune microenvironment might hold great promise for identifying novel molecular targets and improving patient management in the era of immunotherapy (23, 37). In the present study, for the first time, we investigated the immune signatures that are closely related to the early recurrence of stage Ia-b NSCLC after surgery. We estimated diverse leukocyte types in the tumors and identified key immune cell types that associate with recurrence of stage Ia-b NSCLC within 40 months after surgical resection. Moreover, we developed a five immune-related gene panel that was significantly related to relapse-free survival (RFS) in patients with stage Ia-b NSCLC and validated it in multiple independent datasets.

It is believed that immune responses are heterogeneous and require numerous different types of infiltrating immune cells to interact in the tumor microenvironment coordinately (32). The TILs play essential roles during the occurrence and development of cancers and have become the main targets of emerging immunotherapies. Previous studies estimated the composition of TILs by flow cytometry or immunohistochemistry (IHC), which could only quantify a few types of immune cells at a time, thus had limitations in illustrating immune profiles in carcinogenesis. In this study, we adopted a proven in-silico approach using CIBERSORT to resolve 22 immune cell types from tumor transcriptome (17, 29). Among the immune cell types estimated in the TCGA dataset, a high proportion of Tregs, M0 and M1 macrophages in the tumors were associated with a significantly increased probability of tumor recurrence within 40 months after surgery. Conversely, an increased proportion of memory B cells were associated with a lower probability of recurrence (**Figure 1**). Further multivariate analysis revealed that Tregs alone was significantly associated with tumor early recurrence (**Figure 2**). Previous studies have also reported that increased Treg counts were associated with worse overall and relapse-free survival in patients with NSCLC (38–40), breast cancer (41), and hepatocellular carcinoma (42), indicating its critical value in identifying patients with high risk of tumor recurrence. However, in validation cohorts, the significant infiltrating difference was only detected in XYEYY and GSE37745 datasets. The infiltration of Tregs lost its significance in GSE31210, GSE116959 and GSE32863 datasets (**Figure 2**), indicating potential discordance when applying this biomarker in clinic practice.

Despite CIBERSORT permitted a comprehensive analysis of the intra-tumoral immune composition in tumors and identified distinct intra-tumoral immune profiles comprising both positive and negative prognostic immune cell types and had been proved to be effective in evaluating immune profiles in the tumor microenvironment. There are some limitations that need to be addressed. First, CIBERSORT uses a limited set of genes to estimate the 22 immune cell types. Gene expression does not always correlate with protein levels in the cells and differential transcriptional activity among cell types can potentially inflate or deflate the estimation of active or inactive immune cells (43). Second, the localization of immune cells identified could not be evaluated since this is a retrospective study based on tumor transcriptome data and microarray data. In addition, CIBERSORT inference also suffered from statistical collinearity when calculating the TILs (44). These confounding factors might result in biased estimations when validating the predicting value of Tregs in multiple cohorts.

To identify reliable early recurrence predictive biomarkers for stage Ia-b NSCLC after curative surgery, we further investigated gene expression data using LASSO regression analysis. Five immune-related genes, SLFN13, RLTPR, HYDIN, MIR4500HG and TPRG1, were identified to be closely correlated with tumor early recurrence. SLFN13 belongs to Schlafen (SLFN/Slfn) family which has been investigated for their involvement in fundamental cellular processes, including growth regulation, differentiation and control of viral replication. Upregulation of SLFN13 was observed in the differentiation of monocytes to monocyte-derived dendritic cells, indicating a regulating role of SLFN13 in human T cell quiescence (45, 46). RLTPR has been identified as a key regulator of the T-cell receptor signaling pathway. It could selectively upregulate the NF-κB pathway in activated T cells and augment T-cell-receptor-dependent production of interleukin 2 (47). Moreover, Malissen and colleagues reported that RLTPR was a lymphoid cell-specific, actin-uncapping protein essential for co-stimulation *via* CD28 and the development of Tregs. RLTPR deficiency may lead to defective CD28-mediated T cell co-stimulation, resulting in defects in both T and B cell compartments, including a decrease of central memory CD8^+^ T cells, Tregs, and memory B cells (48–50). HYDIN family has been recently identified as novel cancer-associated antigens recognized by adaptive immunity, indicating a potential role in the pathogenesis of cancer (51). Additionally, TPRG1 was also reported to be involved in the regulation of immune response (52).

Our study first evaluated the predictive performance of individual gene biomarkers for lung cancer early recurrence. Each gene can predict tumor early recurrence from many patients, which confirmed the utility of SLFN13, RLTPR, HYDIN, MIR4500HG and TPRG1. Furthermore, by combining these five genes in a panel, we greatly improve the sensitivity without a substantial decline in specificity (**Figure 4G** and **Table 2**). The five gene combination was able to provide a very high sensitivity ranging from 61% to 89% and a specificity ranging from 54% to 85% from training and validation cohorts (**Tables 2**, **3**). Due to the fact that gene expression in individuals might depend on the alterations of different molecular pathways, the use of a multiple gene signature may provide greater utility for predicting when compared with a single gene.

Currently, there is no clinically applicable, unambiguous biomarker signature panel for the early recurrence of stage Ia-b NSCLC after surgery. The current TNM staging system relies significantly on anatomic factors and is limited in its ability to discriminate a subset of patients with stage I disease with a poor survival rate. In fact, for stage Ia-b NSCLCs, tumor size is the only prognostic factor available. For the first time, our study identified five immune-related genes that correlated with cancer early recurrence. Notably, the five-gene panel showed predicting accuracy in all validation datasets, suggesting the potential clinical application for the predicting of early recurrence of stage Ia-b NSCLC.

There are several limitations to our study. First, the number of patients studied was relatively small, although we tried to include as many datasets as possible for more rigorous validation of our biomarkers. Second, gene expression signatures are subject to sampling bias caused by intratumor genetic heterogeneity. The last limitation is the retrospective nature of this study. Prospective studies are needed to further validate its analytical accuracy for predicting cancer early recurrence and testing its clinical utility in the individualized management of stage Ia-b NSCLC.

## Conclusion

Our study demonstrated that the immune microenvironment signatures were closely related to the early recurrence of stage Ia-b NSCLC. The concomitant levels of macrophages and Tregs, in addition to the memory B cells in tumors, indicate substantial risk for tumor early recurrence in patients with stage Ia-b NSCLC. Compared to tumor-infiltrating lymphocytes, the expression of five immune-related genes could be robust biomarkers to predict early recurrence of stage Ia-b NSCLC within 40 months after curative resection.

## Data Availability Statement

The original contributions presented in the study are included in the article/**Supplementary Material**. Further inquiries can be directed to the corresponding author.

## Ethics Statement

The studies involving human participants were reviewed and approved by Institutional Review Board of the Second Xiangya Hospital of Central South University. The ethics committee waived the requirement of written informed consent for participation.

## Author Contributions

Conception and design: CC. Development of methodology: CC, DZ, and QH. Acquisition of data: CC, QW, FW, QL, TY, XW, and YH. Analysis and interpretation of data: CC, DZ, BQ, MP, and QH. Writing, review, and/or revision of the manuscript: CC, DZ, QW, JT, MP, and QH. Administrative, technical, or material support: QH, JT, FW, YH. Study supervision: CC, WL, and FY. All authors contributed to the article and approved the submitted version.

## Funding

This study was funded by the China National Natural Science Foundation (No.81000905 and No.81972638), the Hunan Provincial Natural Science Foundation (No. 14JJ4014, No. 2020SK53419 and No. 2019JJ50953), Hunan Provincial Key Area R&D Program NO. 2019SK2253, CSCO Cancer Research Foundation (CSCO-Y-young2019-034 and CSCO-2019Roche-073), and the Changsha Municipal Natural Science Foundation NO. kq2014246.

## Conflict of Interest

Author QH was employed by company Geneplus-Beijing Institute.

The remaining authors declare that the research was conducted in the absence of any commercial or financial relationships that could be construed as a potential conflict of interest.

## Publisher’s Note

All claims expressed in this article are solely those of the authors and do not necessarily represent those of their affiliated organizations, or those of the publisher, the editors and the reviewers. Any product that may be evaluated in this article, or claim that may be made by its manufacturer, is not guaranteed or endorsed by the publisher.

## References

[1] Chen W Zheng R Baade PD Zhang S Zeng H Bray F. Cancer Statistics in China, 2015. CA Cancer J Clin (2016) 66(2):115–32. 10.3322/caac.21338 26808342

[2] Siegel RL Miller KD Jemal A. Cancer Statistics, 2020. CA Cancer J Clin (2020) 70(1):7–30. 10.3322/caac.21590 31912902

[3] Ramalingam SS Owonikoko TK Khuri FR. Lung Cancer: New Biological Insights and Recent Therapeutic Advances. CA: A Cancer J Clin (2011) 61(2):91–112. 10.3322/caac.20102 21303969

[4] Detterbeck FC Boffa DJ Kim AW Tanoue LT. The Eighth Edition Lung Cancer Stage Classification. Chest (2017) 151(1):193–203. 10.1016/j.chest.2016.10.010 27780786

[5] Uramoto H Tanaka F. Recurrence After Surgery in Patients With NSCLC. Transl Lung Cancer Res (2014) 3(4):242–9. 10.3978/j.issn.2218-6751.2013.12.05 PMC4367696 25806307

[6] Yamashita T Uramoto H Onitsuka T Ono K Baba T So T. Association Between Lymphangiogenesis-/Micrometastasis- and Adhesion-Related Molecules in Resected Stage I NSCLC. Lung Cancer (2010) 70(3):320–8. 10.1016/j.lungcan.2010.02.013 20363046

[7] Boyd JA Hubbs JL Kim DW Hollis D Marks LB Kelsey CR. Timing of Local and Distant Failure in Resected Lung Cancer: Implications for Reported Rates of Local Failure. J Thorac Oncol (2010) 5(2):211–4. 10.1097/JTO.0b013e3181c20080 19901853

[8] Wood DE. National Comprehensive Cancer Network (NCCN) Clinical Practice Guidelines for Lung Cancer Screening. Thorac Surg Clin (2015) 25(2):185–97. 10.1016/j.thorsurg.2014.12.003 25901562

[9] Smith RA Andrews K Brooks D DeSantis CE Fedewa SA Lortet-Tieulent J. Cancer Screening in the United States, 2016: A Review of Current American Cancer Society Guidelines and Current Issues in Cancer Screening. CA: A Cancer J Clin (2016) 66(2):95–114. 10.3322/caac.21336 26797525

[10] Yan TD Black D Bannon PG McCaughan BC. Systematic Review and Meta-Analysis of Randomized and Nonrandomized Trials on Safety and Efficacy of Video-Assisted Thoracic Surgery Lobectomy for Early-Stage Non-Small-Cell Lung Cancer. J Clin Oncol (2009) 27(15):2553–62. 10.1200/JCO.2008.18.2733 19289625

[11] Suzuki K Kadota K Sima CS Nitadori J Rusch VW Travis WD. Clinical Impact of Immune Microenvironment in Stage I Lung Adenocarcinoma: Tumor Interleukin-12 Receptor Beta2 (IL-12rbeta2), IL-7R, and Stromal FoxP3/CD3 Ratio Are Independent Predictors of Recurrence. J Clin Oncol (2013) 31(4):490–8. 10.1200/JCO.2012.45.2052 PMC3731922 23269987

[12] Barlesi F Mazieres J Merlio JP Debieuvre D Mosser J Lena H. Routine Molecular Profiling of Patients With Advanced Non-Small-Cell Lung Cancer: Results of a 1-Year Nationwide Programme of the French Cooperative Thoracic Intergroup (IFCT). Lancet (2016) 387(10026):1415–26. 10.1016/S0140-6736(16)00004-0 26777916

[13] Kadara H Choi M Zhang J Parra ER Rodriguez-Canales J Gaffney SG. Whole-Exome Sequencing and Immune Profiling of Early-Stage Lung Adenocarcinoma With Fully Annotated Clinical Follow-Up. Ann Oncol (2018) 29(4):1072. 10.1093/annonc/mdx062 29688333 PMC6887935

[14] Cancer Genome Atlas Research N. Comprehensive Molecular Profiling of Lung Adenocarcinoma. Nature (2014) 511(7511):543–50. 10.1038/nature13385 PMC4231481 25079552

[15] Chen J Yang H Teo ASM Amer LB Sherbaf FG Tan CQ. Genomic Landscape of Lung Adenocarcinoma in East Asians. Nat Genet (2020) 52(2):177–86. 10.1038/s41588-019-0569-6 32015526

[16] Hirsch FR Scagliotti GV Mulshine JL Kwon R Curran WJ Jr Wu YL. Lung Cancer: Current Therapies and New Targeted Treatments. Lancet (2017) 389(10066):299–311. 10.1016/S0140-6736(16)30958-8 27574741

[17] Gentles AJ Newman AM Liu CL Bratman SV Feng W Kim D. The Prognostic Landscape of Genes and Infiltrating Immune Cells Across Human Cancers. Nat Med (2015) 21(8):938–45. 10.1038/nm.3909 PMC4852857 26193342

[18] Zheng S Luo X Dong C Zheng D Xie J Zhuge L. A B7-CD28 Family Based Signature Demonstrates Significantly Different Prognoses and Tumor Immune Landscapes in Lung Adenocarcinoma. Int J Cancer (2018) 143(10):2592–601. 10.1002/ijc.31764 30152019

[19] Grivennikov SI Greten FR Karin M. Immunity, Inflammation, and Cancer. Cell (2010) 140(6):883–99. 10.1016/j.cell.2010.01.025 PMC2866629 20303878

[20] Bocchialini G Lagrasta C Madeddu D Mazzaschi G Marturano D Sogni F. Spatial Architecture of Tumour-Infiltrating Lymphocytes as a Prognostic Parameter in Resected Non-Small-Cell Lung Cancer. Eur J Cardiothorac Surg (2020) 58(3):619–28. 10.1093/ejcts/ezaa098 32267920

[21] van der Leun AM Thommen DS Schumacher TN. CD8(+) T Cell States in Human Cancer: Insights From Single-Cell Analysis. Nat Rev Cancer (2020) 20(4):218–32. 10.1038/s41568-019-0235-4 PMC7115982 32024970

[22] Lu Z Zou J Li S Topper MJ Tao Y Zhang H. Epigenetic Therapy Inhibits Metastases by Disrupting Premetastatic Niches. Nature (2020) 579(7798):284–90. 10.1038/s41586-020-2054-x PMC8765085 32103175

[23] Li B Cui Y Diehn M Li R. Development and Validation of an Individualized Immune Prognostic Signature in Early-Stage Nonsquamous Non-Small Cell Lung Cancer. JAMA Oncol (2017) 3(11):1529–37. 10.1001/jamaoncol.2017.1609 PMC5710196 28687838

[24] Zhang C Zhang Z Zhang G Zhang Z Luo Y Wang F. Clinical Significance and Inflammatory Landscapes of a Novel Recurrence-Associated Immune Signature in Early-Stage Lung Adenocarcinoma. Cancer Lett (2020) 479:31–41. 10.1016/j.canlet.2020.03.016 32201203

[25] Ettinger DS Wood DE Aggarwal C Aisner DL Akerley W Bauman JR. NCCN Guidelines Insights: Non-Small Cell Lung Cancer, Version 1.2020. J Natl Compr Canc Netw (2019) 17(12):1464–72. 10.6004/jnccn.2019.0059 31805526

[26] Brock MV Hooker CM Ota-Machida E Han Y Guo M Ames S. DNA Methylation Markers and Early Recurrence in Stage I Lung Cancer. N Engl J Med (2008) 358(11):1118–28. 10.1056/NEJMoa0706550 18337602

[27] Zhao M Liu S Luo S Wu H Tang M Cheng W. DNA Methylation and mRNA and microRNA Expression of SLE CD4+ T Cells Correlate With Disease Phenotype. J Autoimmun (2014) 54:127–36. 10.1016/j.jaut.2014.07.002 25091625

[28] Wang H Lu D Liu X Jiang J Feng S Dong X. Survival-Related Risk Score of Lung Adenocarcinoma Identified by Weight Gene Co-Expression Network Analysis. Oncol Lett (2019) 18(5):4441–8. 10.3892/ol.2019.10795 PMC6781564 31611953

[29] Newman AM Liu CL Green MR Gentles AJ Feng W Xu Y. Robust Enumeration of Cell Subsets From Tissue Expression Profiles. Nat Methods (2015) 12(5):453–7. 10.1038/nmeth.3337 PMC4739640 25822800

[30] Ali HR Chlon L Pharoah PD Markowetz F Caldas C. Patterns of Immune Infiltration in Breast Cancer and Their Clinical Implications: A Gene-Expression-Based Retrospective Study. PloS Med (2016) 13(12):e1002194. 10.1371/journal.pmed.1002194 27959923 PMC5154505

[31] Fu H Zhu Y Wang Y Liu Z Zhang J Xie H. Identification and Validation of Stromal Immunotype Predict Survival and Benefit From Adjuvant Chemotherapy in Patients With Muscle-Invasive Bladder Cancer. Clin Cancer Res (2018) 24(13):3069–78. 10.1158/1078-0432.CCR-17-2687 29514839

[32] Chen C Huang X Yin W Peng M Wu F Wu X. Ultrasensitive DNA Hypermethylation Detection Using Plasma for Early Detection of NSCLC: A Study in Chinese Patients With Very Small Nodules. Clin Epigenet (2020) 12(1):39. 10.1186/s13148-020-00828-2 PMC7057485 32138766

[33] Hulbert A Jusue-Torres I Stark A Chen C Rodgers K Lee B. Early Detection of Lung Cancer Using DNA Promoter Hypermethylation in Plasma and Sputum. Clin Cancer Res (2017) 23(8):1998–2005. 10.1158/1078-0432.CCR-16-1371 27729459 PMC6366618

[34] Wick DA Webb JR Nielsen JS Martin SD Kroeger DR Milne K. Surveillance of the Tumor Mutanome by T Cells During Progression From Primary to Recurrent Ovarian Cancer. Clin Cancer Res (2014) 20(5):1125–34. 10.1158/1078-0432.CCR-13-2147 24323902

[35] Dong ZY Zhang C Li YF Su J Xie Z Liu SY. Genetic and Immune Profiles of Solid Predominant Lung Adenocarcinoma Reveal Potential Immunotherapeutic Strategies. J Thorac Oncol (2018) 13(1):85–96. 10.1016/j.jtho.2017.10.020 29127022

[36] Rooney MS Shukla SA Wu CJ Getz G Hacohen N. Molecular and Genetic Properties of Tumors Associated With Local Immune Cytolytic Activity. Cell (2015) 160(1-2):48–61. 10.1016/j.cell.2014.12.033 25594174 PMC4856474

[37] Vargas AJ Harris CC. Biomarker Development in the Precision Medicine Era: Lung Cancer as a Case Study. Nat Rev Cancer (2016) 16(8):525–37. 10.1038/nrc.2016.56 PMC6662593 27388699

[38] Tao H Mimura Y Aoe K Kobayashi S Yamamoto H Matsuda E. Prognostic Potential of FOXP3 Expression in Non-Small Cell Lung Cancer Cells Combined With Tumor-Infiltrating Regulatory T Cells. Lung Cancer (2012) 75(1):95–101. 10.1016/j.lungcan.2011.06.002 21719142

[39] Mony JT Schuchert MJ. Prognostic Implications of Heterogeneity in Intra-Tumoral Immune Composition for Recurrence in Early Stage Lung Cancer. Front Immunol (2018) 9:2298. 10.3389/fimmu.2018.02298 30374348 PMC6196259

[40] Shimizu K Nakata M Hirami Y Yukawa T Maeda A Tanemoto K. Tumor-Infiltrating Foxp3+ Regulatory T Cells Are Correlated With Cyclooxygenase-2 Expression and Are Associated With Recurrence in Resected Non-Small Cell Lung Cancer. J Thorac Oncol (2010) 5(5):585–90. 10.1097/JTO.0b013e3181d60fd7 20234320

[41] Wang L Simons DL Lu X Tu TY Solomon S Wang R. Connecting Blood and Intratumoral Treg Cell Activity in Predicting Future Relapse in Breast Cancer. Nat Immunol (2019) 20(9):1220–30. 10.1038/s41590-019-0429-7 PMC8802768 31285626

[42] Zhou SL Zhou ZJ Hu ZQ Huang XW Wang Z Chen EB. Tumor-Associated Neutrophils Recruit Macrophages and T-Regulatory Cells to Promote Progression of Hepatocellular Carcinoma and Resistance to Sorafenib. Gastroenterology (2016) 150(7):1646–58.e17. 10.1053/j.gastro.2016.02.040 26924089

[43] Kargl J Busch SE Yang GH Kim KH Hanke ML Metz HE. Neutrophils Dominate the Immune Cell Composition in Non-Small Cell Lung Cancer. Nat Commun (2017) 8:14381. 10.1038/ncomms14381 28146145 PMC5296654

[44] Li B Severson E Pignon JC Zhao H Li T Novak J. Comprehensive Analyses of Tumor Immunity: Implications for Cancer Immunotherapy. Genome Biol (2016) 17(1):174. 10.1186/s13059-016-1028-7 27549193 PMC4993001

[45] Puck A Aigner R Modak M Cejka P Blaas D Stockl J. Expression and Regulation of Schlafen (SLFN) Family Members in Primary Human Monocytes, Monocyte-Derived Dendritic Cells and T Cells. Results Immunol (2015) 5:23–32. 10.1016/j.rinim.2015.10.001 26623250 PMC4625362

[46] Yang JY Deng XY Li YS Ma XC Feng JX Yu B. Structure of Schlafen13 Reveals a New Class of tRNA/rRNA- Targeting RNase Engaged in Translational Control. Nat Commun (2018) 9(1):1165. 10.1038/s41467-018-03544-x 29563550 PMC5862951

[47] Park J Yang J Wenzel AT Ramachandran A Lee WJ Daniels JC. Genomic Analysis of 220 CTCLs Identifies a Novel Recurrent Gain-of-Function Alteration in RLTPR (P. Q575E) Blood (2017) 130(12):1430–40. 10.1182/blood-2017-02-768234 PMC5609333 28694326

[48] Roncagalli R Cucchetti M Jarmuzynski N Gregoire C Bergot E Audebert S. The Scaffolding Function of the RLTPR Protein Explains Its Essential Role for CD28 Co-Stimulation in Mouse and Human T Cells. J Exp Med (2016) 213(11):2437–57. 10.1084/jem.20160579 PMC5068240 27647348

[49] Yonkof JR Gupta A Rueda CM Mangray S Prince BT Rangarajan HG. A Novel Pathogenic Variant in CARMIL2 (RLTPR) Causing CARMIL2 Deficiency and EBV-Associated Smooth Muscle Tumors. Front Immunol (2020) 11:884. 10.3389/fimmu.2020.00884 32625199 PMC7314954

[50] Liang Y Cucchetti M Roncagalli R Yokosuka T Malzac A Bertosio E. The Lymphoid Lineage-Specific Actin-Uncapping Protein Rltpr Is Essential for Costimulation via CD28 and the Development of Regulatory T Cells. Nat Immunol (2013) 14(8):858–66. 10.1038/ni.2634 23793062

[51] Laske K Shebzukhov YV Grosse-Hovest L Kuprash DV Khlgatian SV Koroleva EP. Alternative Variants of Human HYDIN Are Novel Cancer-Associated Antigens Recognized by Adaptive Immunity. Cancer Immunol Res (2013) 1(3):190–200. 10.1158/2326-6066.CIR-13-0079 24777681

[52] Liu H Yang Y Ge Y Liu J Zhao Y. TERC Promotes Cellular Inflammatory Response Independent of Telomerase. Nucleic Acids Res (2019) 47(15):8084–95. 10.1093/nar/gkz584 PMC6735767 31294790

