# Supplementary material for: Immune Microenvironment Signatures as Biomarkers to Predict Early Recurrence of Stage Ia-b Lung Cancer
Source: Front Oncol. 2021 Jul 28;11:680287. doi: 10.3389/fonc.2021.680287 (PMC8356052; doi:10.3389/fonc.2021.680287)
Supplement: Supplementary file 1 [file DataSheet_1.docx]

Supplemental Figure S1.

IHC analysis confirmed the enrichment of Tregs in the recurrence group in the XYEYY cohort, while the infiltration of CD4^+^ lymphocytes and CD8^+^ lymphocytes showed no differences between the two groups.


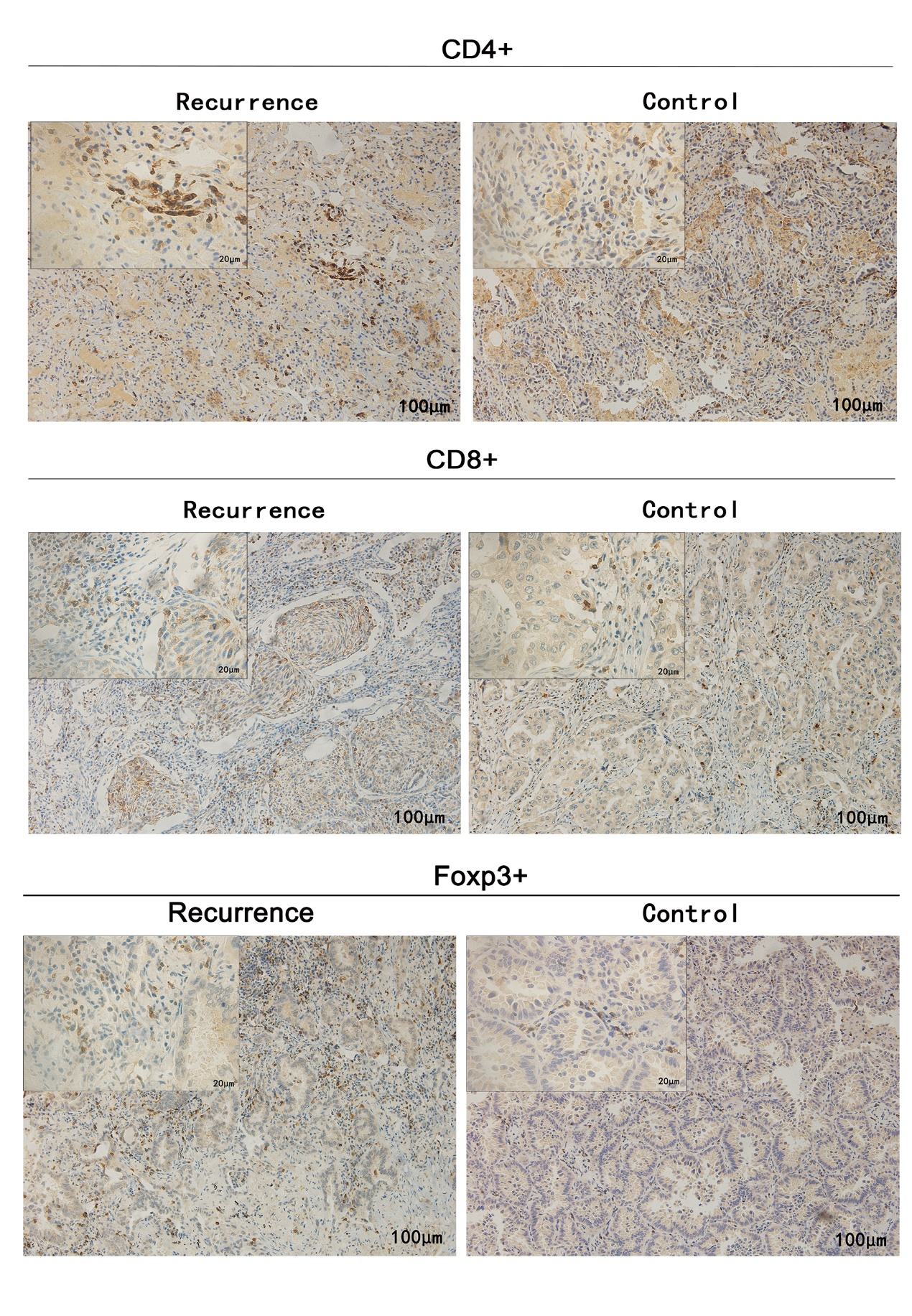


Supplemental Table S1A. Clinical characteristics of patients from XYEYY cohort.

| Patient Characteristics | Recurrence group  (n=42) | Control group  (n=86) | p*-*value |
| --- | --- | --- | --- |
| Age (year, IQR) | 66(49-74) | 65(48-76) | 0.12 |
| Gender (%) |  |  |  |
| Male | 18(42.9%) | 31(36.0%) | 0.48 |
| Female | 24(57.1%) | 55(64.0%) |  |
| Stage-no.(%) |  |  |  |
| Ia | 15(35.7%) | 35(40.7%) | 0.58 |
| Ib | 27(64.3%) | 51(59.3%) |  |
| Recurrence event type |  |  | N/A |
| Locoregional | 26(61.9%) | N/A |  |
| Distant Metastasis | 16(38.1%) | N/A |  |
| Gene mutation events-no*. |  |  |  |
| EGFR | 18(58.1%) | 35(53.8%) | 0.70 |
| BRAF | 0(0.0%) | 2(3.1%) | 0.32 |
| ALK | 3(9.7%) | 5(7.7%) | 0.74 |
| KRAS | 8(25.8%) | 15(23.1%) | 0.77 |
| ERBB2 | 2(6.5%) | 2(3.1%) | 0.44 |
| PIK3CA | 1(3.2%) | 2(3.1%) | 0.97 |
| STK11 | 0(0.0%) | 1(1.5%) | 0.49 |
| Pack-Year (IQR) | 45(20-180) | 42 (40-200) | 0.36 |
| FEV1/FVC (%, IQR) | 76 (65-115) | 79 (60-115) | 0.72 |

*, the gene mutation information could be found in 96 patients in the XYEYY cohort, 31 in the recurrent group and 65 in the control group.

Supplemental Table S1B. Clinical characteristics of patients from GSE31210 dataset.

| Patient Characteristics | Recurrence group  (n=28) | Control group  (n=115) | p*-*value |
| --- | --- | --- | --- |
| Age (year, IQR) | 63(39-71) | 61(34-76) | 0.28 |
| Gender (%) |  |  |  |
| Male | 13(46.4%) | 48(41.7%) | 0.65 |
| Female | 15(53.6%) | 67(58.3%) |  |
| Stage-no.(%) |  |  |  |
| Ia | 11(39.3%) | 85(73.9%) | 0.001 |
| Ib | 17(60.7%) | 30(26.1%) |  |
| Recurrence event type | N/A | N/A | N/A |
| Gene mutation events-no. |  |  |  |
| EGFR | 13(46.4%) | 77(67.0%) | 0.04 |
| ALK | 0(0.0%) | 2(1.7%) | 0.48 |
| KRAS | 3(10.7%) | 10(8.7%) | 0.74 |
| Smoking history* |  |  |  |
| Never | 15(53.6%) | 67(58.3%) | 0.65 |
| Ever | 13(46.4%) | 48(41.7%) |  |

*, smoking status was recorded as Never-smoking or Ever-smoking in GSE31210.

Supplemental Table S1C. Clinical characteristics of patients from GSE32863 dataset.

| Patient Characteristics | Recurrence group  (n=10) | Control group  (n=20) | p*-*value |
| --- | --- | --- | --- |
| Age (year, IQR) | 73(53-78) | 72(47-86) | 0.28 |
| Gender (%) |  |  |  |
| Male | 2(20.0%) | 4(20.0%) | 1.00 |
| Female | 8(80.0%) | 16(80.0%) |  |
| Stage-no.(%) |  |  |  |
| Ia | 4(40.0%) | 10(50.0%) | 0.61 |
| Ib | 6(60.0%) | 10(50.0%) |  |
| Recurrence event type | N/A | N/A | N/A |
| Gene mutation events-no. |  |  |  |
| EGFR | 3(30.0%) | 5(25.0%) | 0.77 |
| KRAS | 5(50.0%) | 8(40.0%) | 0.60 |
| IKB1 | 1(10.0%) | 1(5.0%) | 0.61 |
| Smoking history* |  |  |  |
| Never | 6(60.0%) | 10(50.0%) | 0.61 |
| Current | 4(40.0%) | 10(50.0%) |  |

*, smoking status was recorded as Never or Current in GSE32863.

Supplemental Table S1D. Clinical characteristics of patients from GSE37745 dataset.

| Patient Characteristics | Recurrence group  (n=21) | Control group  (n=20) | p*-*value |
| --- | --- | --- | --- |
| Age (year, IQR) | 69(51-77) | 66(49-75) | 0.19 |
| Gender (%) |  |  |  |
| Male | 12(57.1%) | 11(55.0%) | 0.89 |
| Female | 9(42.9%) | 9(45.0%) |  |
| Stage-no.(%) |  |  |  |
| Ia | 6(28.6%) | 8(40.0%) | 0.44 |
| Ib | 15(71.4%) | 12(60.0%) |  |
| Recurrence event type | N/A | N/A | N/A |
| Gene mutation events-no. | N/A | N/A | N/A |

Supplemental Table S1E. Clinical characteristics of patients from GSE116959 dataset.

| Patient Characteristics | Recurrence group  (n=16) | Control group  (n=12) | p*-*value |
| --- | --- | --- | --- |
| Age (year, IQR) | 65(47-79) | 62(44-76) | 0.34 |
| Gender (%) |  |  |  |
| Male | 13(81.2%) | 9(75.0%) | 0.69 |
| Female | 3(18.8%) | 3(25.0%) |  |
| Stage-no.(%) |  |  |  |
| Ia | 8(50%) | 4(33.3%) | 0.38 |
| Ib | 8(50%) | 8(66.7%) |  |
| Recurrence event type | N/A | N/A | N/A |
| Gene mutation events-no. | N/A | N/A | N/A |

Supplemental Table S2. Primers for qRT-PCR.

| **Gene** | **Forward 5' - 3'** | **Reverse 5' - 3'** |
| --- | --- | --- |
| RLTPR | TGCAGTCCATCAGATCCAAG | GCACCGACTGACACAATTCA |
| SLFN13 | GACGCAGATCCAGAGTTTCC | AAATGTCCTGGTGGAACTGG |
| MIR4500HG | TTGGCTGCGACTCTTACTCA | TCATCTCTGTGGCCTTCCTC |
| HYDIN | CAACATGGCCCAGGATTACG | GCAGGGAGTGTCACATAGC |
| TPRG1 | GGCAAGTTCACCTTCCCT | AGTAGCATAAGGAACTTCAGTGG |
| β-Actin | GCTACGAGCTGCCTGACGG | GATGGAGTTGAAGGTAGTTTCG |
